# Simultaneous acute appendicitis and ectopic pregnancy

**DOI:** 10.4103/0974-2700.44683

**Published:** 2009

**Authors:** Amal Ankouz, Abdelmalek Ousadden, Karim Ibn Majdoub, Ali Chouaib, Khalid Maazaz, Khalid Ait Taleb

**Affiliations:** Department of general surgery, UH Hassan II, Fez, Morocco

**Keywords:** Acute appendicitis, ectopic pregnancy, human chorionic gonadotropin, laparoscopy, laparotomy, sonogram

## Abstract

The acute abdomen in pregnancy is a surgical emergency. Ectopic pregnancy and appendicitis are two causes of acute abdomen in pregnancy. Difficulties in correctly identifying the cause of the pain can be hazardous to the patient and care needs to be taken in obtaining a prompt and accurate diagnosis enabling the most appropriate management. The case presented here underlies the pathogenesis of the simultaneous existence of these two conditions in a patient.

## INTRODUCTION

Ectopic pregnancy and appendicitis are two causes of acute abdomen in pregnancy. The occurrence of ectopic pregnancy and appendicitis during the same pregnancy is unusual and required timely diagnosis and therapy to avoid potentially high maternal morbidity and mortality. Through this case report of concurrent ectopic pregnancy and appendicitis, we describe the unusual occurrence of both these acute conditions happening simultaneously.

## CASE REPORT

A 38-year-old woman, gravida 4, para 3, was admitted to the emergency room at the university hospital of Hassan II. She presented with a 2 day history of extreme right iliac fossa pain. She experienced heavy bleeding for one day. The last reported menstrual period was 10 weeks before presentation. She denied fevers, chills, vomiting, constipation, and diarrhea. Past medical, obstetric, gynaecological, and surgical histories were remarkable only for 3 uncomplicated live births and no history of sexually transmitted diseases or pelvic inflammatory disease. On examination, she was found to have a blood pressure of 120/80 mmHg, a pulse rate of 80 beats/min and a respiratory rate of 20 breaths/min; she was mildly pyrexial at 37.5°C. Abdominal examination revealed tenderness in the right iliac fossa. Gynaecological examination revealed a closed cervix and a right adnexal mass.

Serum level of β-human chorionic gonadotropin (β-hCG) was 3175 IU/ml and complete blood cell count was significant for a leukocyte count of 14,000/mm^3^ with 80% polymorphonuclear leukocytes, hematocrit of 0.30, and a platelet count of 300,000. A pelvic sonogram revealed echogenic mass in the right adnexa measuring 4×5 cm with empty uterine cavity. An abdominal sonogram showed a thickening of the appendix wall associated with a free intraperitoneal fluid.

Diagnoses of appendicitis and ectopic pregnancy were entertained preoperatively, but neither could be excluded by history, physical, laboratory, and radiology examinations.

Because of the uncertainty in diagnosis, emergency exploratory laparotomy was performed through a subumbilical incision, leading to the surprising finding of concurrent ruptured ectopic pregnancy and appendicitis. She was found to have 400 ml of blood free in the pelvis with a perforated right-sided tubal pregnancy and a grossly inflamed appendix. Right salpingectomy, appendectomy and evacuation of blood clot were performed without complications. Post-operatively the patient received intravenous antibiotics; she made an excellent recovery. She was discharged from the hospital in stable condition 3 days later with a prescription for oral antibiotics. Pathologic examination of the resected segment of appendix revealed edema and inflammation of the appendix without abscess formation, consistent with early appendicitis. Pathology of the resected right fallopian tube revealed immature placental tissue and villi consistent with ectopic pregnancy.

## DISCUSSION

Abdominal pain that occurs during pregnancy is challenging to work up because of the broad range of differential diagnoses and distortions of anatomic relationships by the gravid uterus. As the most common cause of surgical pain in pregnant patients, appendicitis is estimated to occur at an incidence of 1 per 2000 pregnancies.[1] Ectopic pregnancy occurs at a frequency of approximately 16 per 1000 patients.[2] However, only 22 such cases have been reported since 1960.[2] It is unknown whether appendicitis is coincidentally associated with ectopic pregnancy. Some authors postulate that ectopic pregnancy may cause an initial contiguous inflammatory reaction in the adjacent appendix which creates a portal for infection in the appendix by normal colonic bacterial flora.[3] The role of assisted conception in the epidemiology of ectopic pregnancies and indirectly in the incidence of this rare dual pathology was reported in the literature.[4] In patient with severe abdominal pain after both in vitro fertilization (IVF) and embryo transfer techniques, appendicitis and ectopic pregnancy should be included in the differential diagnosis.[4]

The diagnosis of acute abdomen in pregnancy is difficult because of the normal changes in physiologic, metabolic, and anatomic states during pregnancy. The white cell count and sedimentation rates can be raised in the gravid state. The appendix localization is changed on account of the gravid uterus. A corollary of this is that lack of definitive findings on sonography, in the presence of high clinical suspicion from a complete history and physical examination, should not preclude a differential diagnosis including appendicitis and ectopic pregnancy in the workup of acute abdomen in a pregnant patient.[5,6]

Because of the uncertainty in diagnosis and to improve the maternal prognosis, emergency exploratory must be practiced. Recently, confirmation of the diagnosis and the management of both ectopic pregnancy and acute appendicitis could have been made by performing a laparoscopy, either microlaparoscopy or classic laparoscopy, prior to proceeding to an open laparotomy as many authors recommend.[5,7,8]

## CONCLUSION

There may be mutual etiologic, pathogenic mechanisms that lead to concurrent development of both ectopic pregnancy and appendicitis through contiguous inflammatory and infectious processes. Therefore one must have the sense not to be unaware of the possibility of simultaneous appendicitis during pregnancy because any delay in making the correct diagnosis can be harmful to the patient.

## References

[1] James DK, Steer PJ, Weiner CP, Gonik B (1995). High risk pregnancy.

[2] Nguyen H, Le K, Le C, Nguyen H (2005). Concurrent ruptured ectopic pregnancy and appendicitis. J Am Board Fam Pract.

[3] Feldman M, Friedman LS, Sleisenger MH (2002). Sleisenger and Fordtran's gastrointestinal and liver disease: Pathophysiology, diagnosis, management.

[4] Daponte A, Spyridakis M, Ioannou M, Vanakara P, Tzovaras G, Hatzitheofilou K (2007). Live birth after laparotomy for concurrent heterotopic pregnancy and appendicitis in a 6 weeks IVF pregnancy. Arch Gynecol Obstet.

[5] Riggs JC, Schiavello HJ, Fixler R (2002). Concurrent appendicitis and ectopic pregnancy: A case report. J Reprod Med.

[6] Andersen B, Nielsen TF (1999). Appendicitis in pregnancy: Diagnosis, management and complications. Acta Obstet Gynecol Scand.

[7] Akman MA, Katz E, Damewood MD, Ramzy AI, Garcia JE (1995). Perforated appendicitis and ectopic pregnancy following in-vitro fertilization. Hum Reprod.

[8] Barnett A, Chipchase J, Hewitt J (1999). Simultaneous rupturing heterotopic pregnancy and acute appendicitis in an in-vitro fertilization twin pregnancy. Hum Reprod.

